# 661 Cyanokit to Use or Not to Use

**DOI:** 10.1093/jbcr/iraf019.290

**Published:** 2025-04-01

**Authors:** Rebecca Coffey, Janie Faris, Kandace Snodgrass, Samuel Mandell

**Affiliations:** Parkland Burn Center; University of Texas Southwestern, Parkland Health; University of Texas Southwestern, Parkland Health; Parkland Burn Center

## Abstract

**Introduction:**

Over 357,500 structure fires occur in the United States annually resulting in about 2,710 civilian deaths. The combustion of plastic, vinyl, wood, silk, and petroleum releases toxic hydrogen cyanide gas. Empiric treatment in the prehospital and hospital setting with hydroxocobalamin has been advocated. Without clear guidelines overuse can occur. The objective of this quality improvement initiative was to evaluate hydroxocobalamin’s use for inhaled cyanide toxicity after implementing a smart order-set and care pathway.

**Methods:**

This is a retrospective, pre (2021-2022) post-group (2023-2024) review of patients with inhalation injury admitted to one verified burn center. Specific criteria for the ordering of hydroxocobalamin for cyanide toxicity were implemented in 2022. Patients without smoke inhalation were excluded. Baseline demographics, laboratory values and medication administration records were evaluated. Analysis of data included: Chi square, Mann Whitney U test, and logistic regression.

**Results:**

120 patients were diagnosed with an inhalation injury and of those 74 patients received hydroxocobalamin prehospital or in the ED (32 patients pre-group, 42 post-group). 46% of the patients received the medication prior to arrival at the burn center. 11 vs 14 patients experienced cardiac arrest in the pre vs post group. 21 (66%) and 20 (48%) of patients in the pre vs post group did not meet criteria to receive the antidote. Administration after 2 hours was the most common reason for inappropriate use in both groups. There was no difference in the mortality rate between groups (p=0.448). There was no difference in hospital or ICU length of stays, or duration of mechanical ventilation. Based on regression analysis, CPR, facial burns, and age were significant predictors for mortality (p=0.002; p= 0.016; p=0.032). The risk of death was increased by 8 times if the patient experienced a cardiac arrest, 4 times if there were facial burns and for every year of age the risk increased by 1 time in patients that received hydroxocobalamin.

**Conclusions:**

Using a smart order-set, along with continued education, will improve compliance with appropriate treatment for suspected cyanide toxicity. The avoidance of inappropriate use of this expensive antidote provides cost savings to the health care system.

**Applicability of Research to Practice:**

Future studies to evaluate the effectiveness of using an evidence-based approach are needed. Smart order-sets can help guide prescribers in ordering and ensuring compliance with guidelines/protocols.

**Funding for the Study:**

N/A

